# Genetic characterization of *Toxoplasma gondii* from pigs from different localities in China by PCR-RFLP

**DOI:** 10.1186/1756-3305-6-227

**Published:** 2013-08-07

**Authors:** Hai-Hai Jiang, Si-Yang Huang, Dong-Hui Zhou, Xiao-Xuan Zhang, Chunlei Su, Shun-Zhou Deng, Xing-Quan Zhu

**Affiliations:** 1 State Key Laboratory of Veterinary Etiological Biology, Key Laboratory of Veterinary Parasitology of Gansu Province, Lanzhou Veterinary Research Institute, Chinese Academy of Agricultural Sciences, Lanzhou, Gansu Province 730046, PR China; 2 College of Animal Science and Technology, Jiangxi Agricultural University, Nanchang, Jiangxi Province 330045, PR China; 3 Department of Microbiology, The University of Tennessee, Knoxville, TN 37996, USA; 4 College of Agriculture, Yanbian University, Yanji, Jilin Province 133002, PR China

**Keywords:** *Toxoplasma gondii*, Toxoplasmosis, Genetic characterization, Pig, China

## Abstract

**Background:**

*Toxoplasma gondii* is a widely prevalent protozoan parasite that causes serious toxoplasmosis in humans and animals. The present study aimed to determine the genetic diversity of *T. gondii* isolates from pigs in Jiangxi, Sichuan, Guangdong Provinces and Chongqing Municipality in China using multilocous polymerase chain reaction-restriction fragment length polymorphism (PCR-RFLP) technology.

**Methods:**

A total of 38 DNA samples were extracted from hilar lymph nodes of pigs with suspected toxoplasmosis, and were detected for the presence of *T. gondii* by semi-nested PCR of B1 gene. The positive DNA samples were typed at 11 genetic markers, including 10 nuclear loci, namely, SAG1, 5′-SAG2 and 3′-SAG2, alternative SAG2, SAG3, BTUB, GRA6, c22-8, c29-2, L358, PK1, and an apicoplast locus Apico.

**Results:**

Twenty-five of the 38 DNA samples were *T. gondii* B1 gene positive. Complete genotyping data for all loci could be obtained for 17 of the 25 samples. Two genotypes were revealed (ToxoDB PCR-RFLP genotypes #9 and #3). Sixteen samples belong to genotype #9 which is the major lineage in mainland China and one sample belongs to genotype #3 which is Type II variant.

**Conclusions:**

To our knowledge, this is the first report of genetic typing of *T. gondii* isolates from pigs in Jiangxi, Sichuan Province and Chongqing Municipality, and the first report of ToxoDB #3 *T. gondii* from pigs in China. These results have implications for the prevention and control of foodborne toxoplasmosis in humans.

## Background

*Toxoplasma gondii* is an obligate intracellular protozoan parasite, infecting all warm-blooded animals and human beings worldwide [1-5]. It is transmitted to humans through consumption of undercooked meat containing *T. gondii* tissue cysts or food or water contaminated by oocysts shed in the feces of infected cats. Toxoplasmosis is one of the main illnesses related to foodborne hospitalizations and deaths [6]. One third of the world population is chronically infected with this parasite [7]. Although *T. gondii* infection in most people appears to be asymptomatic, it may result in life-threatening illness in some immuno-compromised individuals [8].

In China, pigs are the primary livestock raised for human consumption, and they are commonly susceptible to *T. gondii*. This provides the parasite a potential route to transmit the infection via consumption of raw or undercooked meat. *T. gondii* may cause serious diseases such as blindness through consuming raw pork [9].

According to genotypic analysis based on multi-locus approaches, such as PCR-RFLP and microsatellite typing, *T. gondii* strains isolated from humans and animals in North America and Europe have been sorted into 3 clonal lineages, referred to as type I, II, and III [10-12]. A fourth clonal lineage (type 12) in North America was identified in wildlife recently [13]. However, *T. gondii* isolates from South America are characterized with higher genetic diversity [14-16].

China is a big country, but only limited reports concerning genetic characterization of *T. gondii* isolates from pigs are available [17-19]. Such information is still not available for some regions of China especially Jiangxi, Sichuan Provinces and Chongqing Municipality, where swine industry plays an important role in agricultural economy. Thus, the objective of this study is to better understand the genetic diversity of *T. gondii* isolates from pigs in these localities of China.

## Methods

### Sample collection

Hilar lymph nodes were collected, post slaughter, from 38 dead pigs (36 slaughter pigs and 2 sows) with suspected toxoplasmosis during May 2010-March 2013. No ethical approval is required. These pigs came from different geographic regions of China, including 33 from Jiangxi Province, 3 from Sichuan Province, 1 from Chongqing Municipality, and 1 from Guangdong Province, and they all exhibited typical symptoms of toxoplasmosis, which manifest primarily as high fever, dyspnea, subcutaneous hemorrhage, abortion, enlargement and necrosis of liver and spleen.

### Genomic DNA extraction

Genomic DNA was extracted from these hilar lymph nodes using TIANamp Genomic DNA kit (TianGen™, Beijing, China) according to manufacturer’s recommendations. In brief, 30 mg of the hilar lymph nodes were treated with sodium dodecyl sulphate/proteinase K at 56°C for overnight digestion in a thermostat water bath. DNA samples were prepared after purification by silica gel column chromatography and eluted into 50 μl elution buffer. Then, a semi-nested PCR was performed to detect the *T. gondii* B1 gene following the previously described protocol [20].

### Genetic characterization of *T. gondii* isolates

Multilocous polymerase chain reaction-restriction fragment length polymorphism (PCR-RFLP) method [21] was employed to genetically characterize the *T. gondii* isolates from pigs. Firstly, pre-amplification was carried out using a set of mixed external primers in a single reaction. Then 1 μl of the products served as template DNA for nested PCR with internal primers for each marker, respectively. The nested PCR products were digested with restriction endonucleases. The restriction fragments were resolved in 2.5%-3% agarose gel to display single nucleotide polymorphisms (SNPs) using a gel document system (UVP GelDoc-It™ Imaging System, Cambridge, U.K.), and the genotypes of *T. gondii* isolates were finally revealed.

## Results and discussion

Twenty-five of the 38 DNA samples were *T. gondii* B1 gene positive, including 23 from slaughter pigs and 2 from sows. Due to low DNA concentration, only 17 DNA samples presented complete genotyping data. The results of genotyping of these isolates and 9 references were summarized in Table 1. Two genotypes were revealed (ToxoDB PCR-RFLP genotypes #9 and #3). It is interesting that only one genotype (ToxoDB#9) was identified from all 12 *T. gondii* isolates from different districts of Jiangxi Province, which suggests that this genotype is predominantly prevalent in Jiangxi Province and the genetic diversity of *T. gondii* may be low in pigs in this province, although further studies using more samples collected from much broader geographical localities of this province are warranted before a valid conclusion can be drawn. Moreover, ToxoDB#9 was also identified in 4 isolates from 3 localities (Zigong and Ziyang in Sichuan and Rongchang in Chongqing) near the border of Sichuan Province and Chongqing Municipality. This same genotype was previously identified in cats in Beijing Municipality, Guangdong and Anhui Provinces [22-24], and it was also founded in pigs in Guangdong, Henan, Yunnan and Anhui Provinces [17-19,25]. Based on these data, ToxoDB#9 was a predominant lineage prevalent in Mainland China. Interestingly, ToxoDB#9 has been identified in North and Latin America [15,26-28], as well as other Asian countries, such as Sri Lanka, Vietnam [29,30], indicating that it has a worldwide distribution. In the near future, it would be interesting to genotype *T. gondii* isolates from humans in the same localities to see if humans and animals share the same genotypes.

**Table 1:** **Summary of genotyping of *Toxoplasma gondii *isolates from pigs in different geographic regions of China**

**Isolate ID**	**Host**	**Location**	**SAG1**	**5′ + 3′ SAG2**	**Alternative SAG2**	**SAG3**	**BTUB**	**GRA6**	**c22-8**	**c29-2**	**L358**	**PK1**	**Apico**	**Genotype**
GT1	Goat	United States	I	I	I	I	I	I	I	I	I	I	I	Reference, Type I, ToxoDB #10
PTG	Sheep	United States	II/III	II	II	II	II	II	II	II	II	II	I	Reference, Type II, ToxoDB #1
CTG	Cat	United States	II/III	III	III	III	III	III	III	III	III	III	I	Reference, Type III, ToxoDB #2
MAS	Human	France	u-1	I	II	III	III	III	u-1	I	I	III	I	Reference, ToxoDB #17
TgCgCa1	Cougar	Canada	I	I	II	III	III	II	II	u-1	I	u-2	I	Reference, ToxoDB #66
TgCatBr5	Cat	Brazil	I	III	III	III	II	III	I	I	I	u-1	I	Reference, ToxoDB #19
TgWtdSc40	WTD	United States	u-1	II	II	II	II	II	II	II	I	II	I	Reference, Type 12, ToxoDB #5
TgCatBr64	Cat	Brazil	I	I	u-1	III	III	III	u-1	I	III	III	I	Reference, ToxoDB #111
TgRsCr1	Toucan	Costa Rica	u-1	I	II	III	I	III	u-2	I	I	III	I	Reference, ToxoDB #52
TgPSZ30	SP	Zigong, Sichuan	u-1	II	II	III	III	II	II	III	II	II	I	ToxoDB #9
TgPSZ41	SP	Zigong, Sichuan	u-1	II	II	III	III	II	II	III	II	II	I	ToxoDB #9
TgPSY19	SP	Ziyang, Sichuan	u-1	II	II	III	III	II	II	III	II	II	I	ToxoDB #9
TgPCR51	SP	Rongchang, Chongqing	u-1	II	II	III	III	II	II	III	II	II	I	ToxoDB #9
TgPJX1	Sow	Wannian, Jiangxi	u-1	II	II	III	III	II	II	III	II	II	I	ToxoDB #9
TgPJX2	SP	Xinjian, Jiangxi	u-1	II	II	III	III	II	II	III	II	II	I	ToxoDB #9
TgPJX3	SP	Nanchang, Jiangxi	u-1	II	II	III	III	II	II	III	II	II	I	ToxoDB #9
TgPJX4	Sow	Xingguo, Jiangxi	u-1	II	II	III	III	II	II	III	II	II	I	ToxoDB #9
TgPJX5	SP	Xingguo, Jiangxi	u-1	II	II	III	III	II	II	III	II	II	I	ToxoDB #9
TgPJX6	SP	Nanchang, Jiangxi	u-1	II	II	III	III	II	II	III	II	II	I	ToxoDB #9
TgPJX7	SP	Yujiang, Jiangxi	u-1	II	II	III	III	II	II	III	II	II	I	ToxoDB #9
TgPJX8	SP	Yujiang, Jiangxi	u-1	II	II	III	III	II	II	III	II	II	I	ToxoDB #9
TgPJX9	SP	Yujiang, Jiangxi	u-1	II	II	III	III	II	II	III	II	II	I	ToxoDB #9
TgPJX10	SP	Yujiang, Jiangxi	u-1	II	II	III	III	II	II	III	II	II	I	ToxoDB #9
TgPJX11	SP	Yujiang, Jiangxi	u-1	II	II	III	III	II	II	III	II	II	I	ToxoDB #9
TgPJX12	SP	Nanchang, Jiangxi	u-1	II	II	III	III	II	II	III	II	II	I	ToxoDB #9
TgPZS1	SP	Zhongshan, Guangdong	II	II	II	II	II	II	II	II	II	II	I	ToxoDB #3

u-1 and u-2^*^represent unique RFLP genotypes, respectively.

*WTD* White-tailed Deer.

*SP* Slaughter pig.

In this study, another genotype ToxoDB#3 (the type II variant) was identified in a pig in Zhongshan, Guangdong Province. This was the fourth time that ToxoDB#3 was identified in China. Previously, this type was founded from sheep in Qinghai Province [17], from birds in Xinjiang Uygur Autonomous Region [31] and from sparrow in Lanzhou, Gansu Province [32]. This is also the first report of ToxoDB#3 from pigs in China, which indicated that ToxoDB#3 is also a major lineage prevalent in Mainland China.

For Chinese people especially the Han ethnic, pork is the main meat of choice. With the development of the economy in China, the standard of life has greatly improved in recent years. As a result, the pig industry and pork products have been driven by increasing consumption for high quality animal protein. Unlike herbivorous cattle and sheep, pigs are omnivores, they feed on a variety of animal meat and vegetable matter, which increases the chance of contact with cat feces and exposure to *T. gondii*, especially for those free-range pigs. Thus, pigs pose a risk for transmission of toxoplasmosis to human beings.

## Conclusions

The present study has genetically characterized *T. gondii* isolates from pigs in Jiangxi, Sichuan, Guangdong Provinces and Chongqing Municipality. Two genotypes were revealed (ToxoDB PCR-RFLP genotypes #9 and #3), with the genotype #9 as the major lineage in mainland China. To our knowledge, this is the first report of genetic typing of *T. gondii* isolates from pigs in these localities, and the first report of ToxoDB genotype #3 from pigs in China. These findings not only enrich genetic diversity of *T. gondii*, but also have implications for the prevention and control of foodborne toxoplasmosis in humans.

## Competing interests

The authors declare that they have no competing interests.

## Authors’ contributions

XQZ and CS conceived and designed the study, and critically revised the manuscript. HHJ, SYH and SZD performed the experiments, analyzed the data and drafted the manuscript. DHZ and XXZ helped in study design, study implementation and manuscript revision. All authors read and approved the final manuscript.
